# Optimization of a rapid method for screening drugs in blood by liquid chromatography tandem mass spectrometry

**DOI:** 10.1515/almed-2023-0154

**Published:** 2023-11-23

**Authors:** Alba M. Rodrigo Valero, Oscar Quintela Jorge, Begoña Bravo Serrano, Sara Ayuso Tejedor

**Affiliations:** Departamento Madrid, Servicio de Química, Instituto Nacional de Toxicología y Ciencias Forenses (INTCF), Madrid, Spain

**Keywords:** screening, removal of phospholipids, mass spectrometry, protein precipitation, blood

## Abstract

**Objectives:**

In the recent years, liquid chromatography with tandem mass spectrometry has gained popularity in laboratories. This technique has a higher specificity, detects different analytes from a single specimen, measures analytes in distinct matrices, and substantially reduce analytical interference, with respect to immunoassay. The processing and preparation of biological samples are crucial in chromatography. Interferences in blood testing are usually caused by the presence of phospholipids and proteins. The main objective of this study was to improve analytical processes for drug screening by LC-MS/MS using a novel blood sample preparation method based on protein precipitation and removal of phospholipids.

**Methods:**

An evaluation was performed of a new method for the preparation of blood samples based on protein precipitation and removal of phospholipids by LC-Q-q-LIT.

**Results:**

Limit of detection, limit of quantification and measurement range were determined for 56 molecules. The results of 11 cases were compared with those obtained using standard blood collection methods and instruments.

**Conclusions:**

The novel blood preparation and testing method based on LC-Q-q-LIT, a more sensitive technique, has demonstrated to yield comparable results to traditional methods. In addition, this new technique reduces turnaround time and costs.

## Introduction

In the last 10–15 years, liquid chromatography-tandem mass spectrometry has gained popularity in clinical laboratories [1, 2]. Benefits of this analytical technique include a higher specificity, the possibility of measuring different analytes in a single sample injection, measuring analytes in different matrices, and a dramatic reduction of analytical interferences, as compared to immunoassay [3]. Notably, immunoassay is still used in many laboratories as the gold-standard method for screening drugs in blood. However, positive results require confirmation by chromatography-tandem mass spectrometry [4].

In the field of clinical and forensic toxicology, the compounds to be detected are unknown *a priori*. In these cases, immunoassays are insufficient, as they screen for a limited number of potentially present substances (or substance families) and lack the required sensitivity and selectivity. Therefore, more effective laboratory strategies are necessary (Systematic Toxicological Analysis, STA) to be able to identify hundreds of relevant toxic substances in biological samples and ensure a correct interpretation of results [5]. This strategy is structured into two stages. Firstly, screening is performed for some drugs and medicines in urine and blood by immunoassay. Secondly, the results obtained in the first stage are confirmed via extended screening for substances that were not included in the first stage. The main analytical techniques used include: HS-GC-FID, GC-MS, GC-MS/MS, LC-MS/MS, with the latter including high-resolution mass spectrometry (LC-HR-MS).

The processing and preparation of biological samples is crucial in chromatography. Their relevance stems from the need to concentrate or remove the different components of the sample that may interfere with results. This step ensures appropriate chromatographic separation and efficacy. The aim of the preanalytical stage, including sample preparation, is to concentrate target substances while removing potential endogenous interfering substances from the biological matrix. The presence of proteins, lipids, salts and cellular components may interfere with the measurement of the analyte under study and condition chromatography performance. The main sample preparation procedures include filtration, centrifugation, dilution, protein precipitation, liquid–liquid extraction (LLE) and solid-phase extraction (SPE). The two latter are the most common techniques for blood and urine testing in clinical toxicology [6]. Despite the advantages of these procedures, most of them may cause a partial component loss. In addition, not all methods eliminate interfering substances completely. The main interfering substances when screening for drugs in blood are phospholipids and proteins. Therefore, sample preparation should be aimed at reducing their presence as much as possible. For this reason, we suggest integrating a special sample (serum or plasma) preparation procedure combining protein precipitation and removal of phospholipids in the preanalytical stage instead of traditional SPE and LLE extractions [7] as it has some advantages. Firstly, it reduces turnaround time, since it is a less time-consuming method. Secondly, being less selective, this technique favors the detection of a higher number of components, as compared to traditional techniques. Its lower selectivity is due to the fact that analytes with very different chemical structures and/or polarity are not removed, as it does occur during SPE and LLE.

The main objective of this study was to improve STA based on LC-MS/MS by using a novel method. This method integrates a new step in blood sample preparation that involves protein precipitation and removal of phospholipids. Performance for qualitative and quantitative analysis of the selected molecules was assessed in terms of limit of detection, and linearity, precision and accuracy, respectively. Likewise, we evaluated its applicability through the analysis of 11 real-life samples received in the INTCF in Madrid. Results were compared with those obtained with traditional extraction methods and instruments.

## Materials and methods

### Reagents, stock and working solutions

The following deuterated internal standards were obtained from Cerilliant^®^ (Round Rock, TX, USA): clomipramine-d3, ketamine-d4, oxazepam-d5, pseudoephedrine-d3, risperidone-d4, salbutamol-d3, tramadol-d3 and zolpidem-d6. LC-MS acetonitrile, LC-MS formic acid, 0.1 % (v/v) formic acid solution in water and 0.1 % (v/v) formic acid solution in LC-MS grade acetonitrile were obtained from Fisher Scientific^®^ (Waltham, MA, USA). 1 M ammonium formate was obtained from Honeywell^®^ (Charlotte, NC, USA) and 0.1 M ZnSO_4_ from Panreac Química S.L.U (Castellar del Vallès, Barcelona, Spain). Deionize water was obtained using a Millipak^®^ filter from Merck-Millipore^®^ (Burlington, MA, USA) formed by a 0.22 µm filtration membrane.

Regarding working solutions, the 0.1 M ZnSO_4_ solution was used for cell lysis and the LC-MS grade acetonitrile (ACN) solution acidified with formic acid (FA) 1 % (v/v) was used for protein precipitation. In addition, the solution of deuterated internal standards was prepared at the known concentration of 0.1 mg/L for analyte quantification. Finally, the mixture for sample reconstitution was also prepared with 90 % water and 10 % ACN, both acidified with 0.1 % (v/v) FA.

The 56 drugs under study were grouped according to their therapeutic group in the following way to facilitate the experimental work; group 1: clobazam, clotiazepam, flunitrazepam, flurazepam, nitrazepam, prazepam, group 2: midazolam, oxazepam, temazepam, tetrazepam, triazolam, zolpidem, group 3: bentazepam, chlordiazepoxide, diazepam, lorazepam, lormetazepam, nordiazepam, group 4: amitriptyline, citalopram, clomipramine, imipramine, paroxetine, sertraline, group 5: nefazodone, nortriptyline, reboxetine, trimipramine, venlafaxine, group 6: maprotiline, mianserin, trazodone, group 7: clonazepam, phenytoin, group 8: amoxapine, buflomedil, clothiapine, codeine, dobutamine, group 9: buspirone, metoclopramide, metronidazole, ofloxacin, quetiapine, telmisartan, group 10: clozapine, levopromazine, loxapine, risperidone, thioproperazine, thioridazine and group 11: 7-aminoclonazepam, N-desalkylflurazepam, zuclopenthixol, chlorprothixene and haloperidol. Each of the 11 solutions described were prepared at concentrations of 2, 0.2, 0.04 and 0.004 μg/mL. These substances were selected according to their prevalence in the intoxications received and the availability of reference standards in the Chemistry Laboratory of INTCF-Madrid.

### Preparation of blood samples

The blank blood used for sample preparation is routinely acquired by INTCF-Madrid from the Blood Transfusion Center from material that, due to certain characteristics cannot be used for medical purposes. A sample of blank blood is processed along with other samples to verify the absence of drugs in it.

The preparation of blood samples was carried out by spiking the analytes selected in human blood, at concentrations of 1, 2, 5, 10, 25, 50, 100, 250, 350, 500, 750 and 1000 ng/mL for the 56 drugs under study, using the previously prepared solutions.

### Protein precipitation and removal of phospholipids

The preparation of samples based on protein precipitation and removal of phospholipids include the following steps: firstly, cellular lysis of 100 µL blood is performed to which 50 µL of the ZnSO_4_ 0.1 M solution is added. After 5 min of resting, 50 µL of the internal standard are added, and protein precipitation is performed by addition and subsequent centrifugation (5 min at 14,000 rpm) of 650 µL of the 1 % (v/v) AF in ACN. The supernatant is loaded on a Phree Phospholipid plate from Phenomenex^®^ (Torrance, CA, USA) and a positive pressure of 10 psi is applied for 5 min approximately. Then, the solution is evaporated to dryness and reconstituted with 200 µL of MP. Next, the solution is filtered by centrifugation (5 min at 14,000 rpm) in an Eppendorf tube with a 0.22 µm pore size membrane filter.

### Instrument

The LC-MS/MS method used utilizes of an ExionLC UHPLC coupled to a Triple Quad 6500+ (QTRAP 6500+) from AB Sciex^®^ (Framingham, MA, USA) consisting of a hybrid triple quadrupole enabling the third quadrupole to function as a linear ion trap (LC-Q-q-LIT). The LIT function provides several enhanced operating modes. Thus, spectra are rapidly acquired in a short period of time and are significantly more intense than spectra acquired using a comparable standard quadrupole method. As a result, sensitivity increases and spectral quality is improved.

Samples were analyzed using a new multiple reaction monitoring-information data acquisition-enhanced product ion (MRM-IDA-EPI) scanning technique that includes the 56 substances under study. For that purpose, we used Analyst^®^ and MultiQuant^®^ software from Sciex.

#### Parameters of high-resolution liquid chromatography

The liquid chromatograph includes a degasser, autosampler, column oven, and two binary pumps.

The MPs used are a mixture of 2 mM ammonium formate in water with 0.2 % (v/v) formic acid for MP A and 2 mM of ammonium formate in ACN with 0.2 % (v/v) formic acid for MP B. The concentration gradient begins with 10 % (v/v) of MP B during the first 0.5 min that increases linearly to 50 % of MP B at 12 min, 90 % of MP B at 14 min and up to 98 % at 15.5 min and then descends to initial conditions at 17.5 min and remains constant for 2.5 additional minutes. The total duration of the experiment is 19.8 min, maintaining a constant flow rate of 0.3 mL/min.

The sample injection volume is 2 µL, and the temperature of the autosampler is 12 °C.

Compound separation is performed using Kinetex 2.6 µm biphenyl 100 Å (100 × 2.1 mm) column from Phenomenex^®^ (Torrance, CA, USA). Column oven temperature is 30 °C.

#### Parameters of mass spectrometry

A mass spectrometer consists of a hybrid triple quadrupole with two preliminary vacuum pumps and a compressed air and nitrogen source coupled to an IonDrive Turbo V ion source that uses the TurboIonSpray probe.

MS conditions are as follows: curtain gas, 35 psi; collision gas, intermediate; IS voltage, 5500 V; ion source temperature, 450 °C; ionization gas 1, 50 psi and ionization gas 2, 60 psi. The MS was set in positive ionization mode for all compounds. The ion trap works when signal intensity exceeds 6,000 counts per second (cps) for ions with an *m*/*z* ranging from 100 to 1,250.

Two transitions were used for the analysis of each analyte, whereas internal standards were analyzed using one transition. The MRM detection window is 60 s and the scan time is 0.4 s. The duration of the experiment was 19.8 min, with a duration of 0.8 s per cycle. Table 1 summarizes retention time, transitions, and specific characteristics of the 56 compounds, including declustering potential (DP), collision energy (CE), and collision cell exit potential (CXP).

**Table 1:** LOD, LLOQ, measurement range, retention time and conditions of MS/MS for the 56 analytes analyzed on Sciex QTRAP 6500+.

Analyte	LOD, ng/mL	LLOQ, ng/mL	Measurement range	Retention time, min	Q1 mass, Da	Q3 mass, Da	DP, V	CE, V	CXP, V	Internal standard
7-Aminoclonazepam	2	5	5–750	6.41	286.1	222.22	70	29	10	Oxazepam-d5
						121.11	60	35	10	
Amitriptyline	5	10	10–1,000	12.19	278.12	91.15	60	15	10	Zolpidem-d6
						191.14	60	15	10	
Amoxapine	1	2	2–750	10.34	314.1	271.1	60	20	10	Oxazepam-d5
						193.1	60	20	10	
Bentazepam	1	5	5–1,000	9.69	297	269.1	60	35	10	Oxazepam-d5
						166.1	60	35	10	
Buflomedilo	1	1	1–1,000	7.84	308.2	237.1	60	50	10	Oxazepam-d5
						195.1	60	50	10	
Buspirone	1	2	2–750	9.18	386.3	122.1	41	25	10	Oxazepam-d5
						265.2	41	25	10	
Citalopram	2	10	10–1,000	10.37	324.94	109.1	60	35	10	Oxazepam-d5
						262.1	91	27	10	
Clobazam	1	1	1–1,000	12.95	301.07	259.18	76	27	10	Risperidona-d4
						224.2	50	46	3	
Clomipramine	2	10	10–1,000	13.09	315.16	86.1	60	50	10	Clomipramina-d3
						242.07	60	50	10	
Clonazepam	2	5	5–1,000	11.96	315.9	270	36	33	16	Oxazepam-d5
						214.1	100	51	14	
Chlordiazepoxide	1	5	5–1,000	8.11	300.16	282.1	60	35	10	Oxazepam-d5
						227.18	60	35	10	
Chlordiazepoxide	2	10	10–750	12.8	316.1	231.1	60	35	10	Risperidona-d4
						221.1	60	35	10	
Clotiapine	1	1	1–750	11.84	344.1	287	60	35	10	Oxazepam-d5
						255.11	60	35	10	
Clotiazepam	2	5	5–1,000	13.25	319.1	291.1	60	50	10	Risperidona-d4
						218.17	60	50	10	
Clozapine	1	10	10–750	8.19	327.1	270.1	60	35	10	Risperidona-d4
						192.16	46	57	8	
Codeine	1	2	2–1,000	4.6	300.3	215.2	66	33	12	Oxazepam-d5
						165.09	120	55	8	
Desalkylflurazepam	2	5	5–1,000	12.03	288.94	226.15	66	50	12	Oxazepam-d5
						140	60	35	10	
Diazepam	1	5	5–750	13.39	284.99	154	60	35	10	Oxazepam-d5
						193.09	60	38	3	
Dobutamine	1	1	1–750	5.93	302.2	137.1	36	31	4	Oxazepam-d5
						107.08	36	31	4	
Phenytoin	10	10	10–1,000	10.6	253.1	104.1	100	30	10	Zolpidem-d6
						182.1	61	23	20	
Flunitrazepam	1	10	10–750	12.91	314	268.1	60	35	10	Clomipramina-d3
						239.14	65	49	3	
Flurazepam	1	10	10–750	10.1	388	315.1	71	25	10	Clomipramina-d3
						287.12	50	40	3	
Haloperidol	1	1	1–1,000	11.04	376.15	165.12	111	37	22	Oxazepam-d5
						123.05	61	51	14	
Imipramine	1	10	10–1,000	11.79	281.2	86.1	76	25	14	Oxazepam-d5
						193.1	76	25	14	
Levomepromazine	1	2	2–1,000	12.34	329.2	100.1	60	35	10	Risperidona-d4
						242.15	60	35	10	
Lorazepam	2	5	5–750	11.2	320.95	274.98	60	50	10	Oxazepam-d5
						229.17	50	41	10	
Lormetazepam	1	2	2–750	12.79	334.97	288.97	60	35	10	Oxazepam-d5
						177.19	50	59	8	
Loxapine	1	2	2–500	10.93	328.08	271.07	51	31	16	Zolpidem-d6
						193.19	41	59	10	
Maprotiline	1	10	10–750	11.99	278.2	250.2	60	35	10	Zolpidem-d6
						191.2	100	49	12	
Metoclopramide	1	10	10–750	6.49	300.2	184.1	60	41	12	Oxazepam-d5
						227.2	56	25	10	
Metronidazol	1	2	2–750	3.51	171.81	128.1	60	20	10	Oxazepam-d5
						82.03	60	20	10	
Mianserin	1	2	2–1,000	10.48	265.12	208.04	60	35	10	Zolpidem-d6
						193.2	60	35	10	
Midazolam	1	2	2–750	9.87	325.98	291.1	60	35	10	Oxazepam-d5
						249.06	60	35	10	
Nefazodone	1	1	1–750	13.06	470.2	274	60	35	10	Oxazepam-d5
						246.2	60	35	10	
Nitrazepam	2	10	10–1,000	11.19	282.1	236.2	60	35	10	Salbutamol-d3
						180.2	126	51	10	
Nordiazepam	1	5	5–750	11.27	270.99	140	60	35	10	Oxazepam-d5
						208.14	60	39	10	
Nortriptyline	1	2	2–750	11.8	264.17	191.16	76	29	14	Oxazepam-d5
						233.12	70	19	14	
Ofloxacin	1	10	10–750	5.5	362.1	261.1	60	35	10	Oxazepam-d5
						318.15	60	35	10	
Oxazepam	5	10	10–750	10.94	287.03	269.2	56	21	32	Oxazepam-d5
						241.11	96	33	14	
Paroxetine	2	25	25–1,000	11.37	330.2	192	91	39	16	Zolpidem-d6
						135.07	91	39	16	
Prazepam	1	2	2–750	14.91	325.07	271.14	60	35	10	Risperidone-d4
						165.13	60	35	10	
Quetiapine	1	1	1–750	9.54	384.13	221.14	96	49	12	Oxazepam-d5
						253.06	60	35	10	
Reboxetine	5	10	10–750	10.29	314.17	176.1	126	43	26	Oxazepam-d5
						91.09	40	39	10	
Risperidone	1	5	5–1,000	8.49	411	191.3	56	27	12	Risperidone-d4
						163.1	56	27	12	
Sertraline	1	10	10–1,000	12.59	306.1	275.1	66	17	14	Zolpidem-d6
						159.02	30	33	10	
Telmisartan	1	5	5–750	12.84	515.2	497.27	100	50	12	Oxazepam-d5
						276.2	60	35	10	
Temazepam	2	10	10–750	12.52	301.2	255.2	101	33	8	Salbutamol-d3
						283.18	76	19	10	
Tetrazepam	5	10	10–750	11.3	288.93	225	60	35	10	Oxazepam-d5
						197.2	106	43	10	
Thioproperazine	2	5	5–1,000	11.3	447	141.2	61	55	10	Risperidone-d4
						319.06	61	55	10	
Thioridazine	1	5	5–1,000	14.31	371.1	126.2	106	33	10	Clomipramina-d3
						258.11	106	33	10	
Trazodone	1	5	5–750	9.3	372.09	176.13	60	35	10	Zolpidem-d6
						148.04	81	48	10	
Triazolam	1	5	5–750	11.95	343.1	308.2	65	39	3	Oxazepam-d5
						239.2	60	35	10	
Trimipramine	1	2	2–750	12.43	295.2	100.1	60	35	10	Oxazepam-d5
						208.15	60	35	10	
Venlafaxine	1	10	10–750	8.09	278	260.3	60	20	10	Oxazepam-d5
						121.05	60	20	10	
Zolpidem	5	10	10–750	8.5	308.1	235.2	60	20	10	Zolpidem-d6
						263.13	60	20	10	
Zuclopentixol	10	25	25–750	11.8	401.11	356.18	60	20	10	Zolpidem-d6
						271.15	60	20	10	
Clomipramine-d3	–	–	–	13.09	318	89.2	60	35	10	
Ketamine-d4	–	–	–	6.53	241.9	129.1	60	30	10	
Oxazepam-d5	–	–	–	10.94	292.13	246.1	96	21	10	
Pseudoephedrine-d3	–	–	–	3.4	169.1	151	60	20	10	
Risperidone-d4	–	–	–	8.49	415.24	195.14	60	35	10	
Salbutamol-d3	–	–	–	2.54	243.04	151.12	56	27	20	
Tramadol-d3	–	–	–	6.84	268	58	60	35	10	
Zolpidem-d6	–	–	–	8.5	314.32	235.2	61	47	4	

The following parameters were evaluated for substance verification: presence of a peak at the appropriate retention time; existence of the two MRM transitions per substance; and identification of substance spectrum through the use of a “spectral library” of compounds.

### Acceptance criteria

The LOD of each analyte was established by analysing different drugs at concentrations of 1, 2, 5, 10, 25 and 50 ng/mL. The lowest concentration with a signal:noise ratio ≥3:1 was established as the LOD. The LLOQ was established using the same method, but with a signal:noise rate ≥10:1. For such purpose, samples were analyzed in triplicate.

With regard to calibration, the linearity of all substances was evaluated from 1 ng/mL (CAL 1) to 1,000 (CAL 12) ng/mL. The quadratic regression model was used for all substances, except for metoclopramide, 7-aminoclonazepam and zuclopenthixol, which followed a linear regression model. In line with previous studies [8, 9], the acceptance criteria for all calibration lines included a coefficient of determination (*r*2) > 0.99; a coefficient of variation (CV) <±20 % at all data points along the curve; and a calibration curve composed of at least seven points.

### Sample analysis

To evaluate the application of the new sample preparation method, 11 blood samples were analyzed with the new method on a QTRAP 6500+ analyzer. These cases had been previously analyzed using traditional sample preparation methods, involving at least a SP and LLE, and using LC-DAD, GC-MS, LC-MS/MS and immunoassay techniques. For some substances, special extractions and/or techniques were also used, including GC-MS/MS and LC-HR-MS (Q Exactive Orbitrap). Finally, the results obtained with traditional extractions and methods were compared against the results of the new method under study.

## Results

Table 1 shows results for LOD, LLOQ and measuring range for all the substances analyzed on QTRAP 6500+. All outlined substances met our acceptance criteria.

Ten of the eleven cases selected were positive, whereas a case was negative. Six of the eleven samples analyzed with the new method were positive. In contrast, five samples were negative, because the drugs in the four positive cases (paracetamol, naproxen, metamizole and benzoylecgonine) were not included in the new method. In the six positive cases, the two methods detected the same substances, except for sertraline in case 11. In contrast, the new method detected lorazepan in case 1. Concentrations were calculated using the calibration curve of each drug and the most appropriate internal standard, as detailed in Table 1. The results obtained were comparable, except for quetiapine in case 1 and diazepam in case 11. Results are shown in Table 2.

**Table 2:** Comparison of the substances detected by the different techniques and methods.

Case number	Traditional method and immunoassay, LC-DAD, GC-MS and LC-MS/MS analysis		New method and QTRAP 6500+ analysis	
1	Lorazepam	ND	Lorazepam	15.4 ng/mL
	Quetiapine	60 ng/mL	Quetiapine	2.6 ng/mL
2	Lorazepam	50 ng/mL	Lorazepam	72.4 ng/mL
5	Codeine	13.3 ng/mL	Codeine	18.1 ng/mL
9	Diazepam	<10 ng/mL	Diazepam	<5 ng/mL
10	Diazepam	40 ng/mL	Diazepam	49.2 ng/mL
11	Metoclopramide	<100 ng/mL	Metoclopramide	40.7 ng/mL
	Haloperidol	8 ng/mL	Haloperidol	1 ng/mL
	Trazodone	300 ng/mL	Trazodone	187 ng/mL
	Diazepam	200 ng/mL	Diazepam	31.9 ng/mL
	Sertraline	100 ng/mL	Sertraline	ND

ND, not detected.

## Discussion

A new sample preparation method was developed based on protein precipitation, removal of phospholipids, and the use of more sensitive techniques, such as LC-Q-q-LIT. Comparable results were obtained for 56 substances with respect to the traditional method, as the two methods virtually detected the same substances. However, this is a preliminary study. A more thorough validation process that evaluates the extraction performance, carryover effect, and matrix effect in all the drugs and internal standards included in this study. Further research is also needed to evaluate drug quantification using the new methodology. The main advantage of the new method is the reduction of time and costs, as compounds of different characteristics can be detected in a single sample, with a high sensitivity and selectivity, and in a shorter time. These characteristics are a considerable advantage, especially in clinical samples.

A limitation of this study was the inability to detect sertraline, and the limited number of substances analyzed. However, this is a good starting point for the future implementation of this new method, especially in clinical samples such as cases of chemical submission, which require rapid testing and a high sensitivity and specificity.
